# Chlamydia trachomatis Lipopolysaccharide Evades the Canonical and Noncanonical Inflammatory Pathways To Subvert Innate Immunity

**DOI:** 10.1128/mBio.00595-19

**Published:** 2019-04-23

**Authors:** Chunfu Yang, Michael Briones, Janice Chiou, Lei Lei, Michael John Patton, Li Ma, Grant McClarty, Harlan D. Caldwell

**Affiliations:** a Laboratory of Clinical Immunology and Microbiology, National Institute of Allergy and Infectious Diseases, National Institutes of Health, Bethesda, Maryland, USA; b Department of Medical Microbiology, University of Manitoba, Winnipeg, Manitoba, Canada; University of California, Irvine; Duke University School of Medicine; University of Arkansas for Medical Sciences; University of California, Irvine

**Keywords:** caspase-11, chlamydia, inflammation, lipopolysaccharide, TLR4

## Abstract

Chlamydia trachomatis is the most common bacterial cause of sexually transmitted infections. *C. trachomatis* sexually transmitted infections are commonly asymptomatic, implying a pathogenic strategy for the evasion of innate inflammatory immune responses, a paradox as the *C. trachomatis* outer membrane contains lipopolysaccharide (LPS), a known potent agonist of inflammatory innate immunity. Here, we studied the ability of chlamydial LPS to activate the proinflammatory canonical and noncanonical inflammasome pathways in mouse bone marrow-derived macrophages (BMDM). We show, in comparison to Escherichia coli LPS, that *C. trachomatis* LPS-treated BMDM produce significantly less IL-6, TNF, and type I interferon mRNA, indicating that downstream signaling through the canonical TLR4 myddosome and triffosome pathways was blocked. We confirmed this in *C. trachomatis* LPS-treated BMDM by showing the lack of NF-κB and IRF3 phosphorylation, respectively. Interestingly, *C. trachomatis* LPS bound CD14 and promoted its endocytosis; however; it did not promote efficient TLR4/MD-2 dimerization or endocytosis, known requirements for myddosome and triffosome signaling pathways. We further found that transfection of BMDM with *C. trachomatis* LPS did not cause pyroptotic cell ballooning, cytotoxicity, or IL-1β secretion, all characteristic features of noncanonical inflammasome activation. Western blotting confirmed that cytosolic *C. trachomatis* LPS failed to signal through caspase-11, as shown by the lack of gasdermin D, caspase-1, or IL-1β proteolytic cleavage. We propose that chlamydiae evolved a unique LPS structure as a pathogenic strategy to avoid canonical and noncanonical innate immune signaling and conclude that this strategy might explain the high incidence of asymptomatic infections.

## Abstract (precis)

Chlamydia trachomatis is the most common bacterial cause of sexually transmitted infections (STI). *C. trachomatis* STI are commonly asymptomatic, implying a pathogenic strategy for the evasion of innate inflammatory immune responses, a paradox as the *C. trachomatis* outer membrane contains lipopolysaccharide (LPS), a known potent agonist of inflammatory innate immunity. Here, we found that *C. trachomatis* LPS is not capable of engaging the canonical TLR4/MD-2 or noncanonical caspase-11 inflammatory pathways. The inability of *C. trachomatis* LPS to trigger innate immunity inflammatory pathways is related to its unique fatty acid structure. Evolutionary modification of the LPS structure likely evolved as a pathogenic strategy to silence innate host defense mechanisms. The findings might explain the high incidence of asymptomatic chlamydial genital infection.

## INTRODUCTION

Chlamydia trachomatis is a Gram-negative obligate intracellular bacterial pathogen of humans that infects ocular and genital epithelium, causing blinding trachoma and sexually transmitted disease which afflict hundreds of millions of people globally. C. trachomatis sexually transmitted infections (STI) are commonly asymptomatic and in women can lead to severe sequelae such as pelvic inflammatory disease (PID), tubal factor infertility, and ectopic pregnancy (1, 2). The reason for the high incidence of asymptomatic infection remains elusive and is paradoxical as the *C. trachomatis* outer membrane contains lipopolysaccharide (LPS), a known potent agonist of inflammatory innate immunity. Therefore, we have investigated whether chlamydial LPS is capable of functioning as a proinflammatory pathogen-associated molecular pattern (PAMP).

C. trachomatis is distinguished by a biphasic developmental growth cycle that modulates between a small metabolically inactive infectious elementary body (EB) and a larger metabolically active noninfectious reticulate body (RB) (3). The chlamydial outer membrane of EBs and RBs contains LPS. Chlamydial LPS is characterized by a group-specific antigenic epitope composed of a trisaccharide of 3-deoxy-d-manno-oct-2-ulosonic acid (Kdo) residues with a unique alpha-Kdo-(2→8)-alpha-Kdo-(2→4)-alpha-Kdo linkage and a penta-acyl lipid A (4–6) and is essential for secondary differentiation of RB to infectious EB (7). *C. trachomatis* LPS is significantly (10- to 100-fold) less stimulatory than enteric LPS in activating proinflammatory signaling by cultured human epithelial cells (8–10). This weak proinflammatory signaling response requires CD14-TLR4 interaction (8, 9). However, the molecular basis for the poor proinflammatory signaling response despite the CD14-TLR4 binding interaction is unknown.

LPS is the prototypical PAMP recognized by host innate immunity pattern recognition receptors (PRR) that activates the canonical TLR4 proinflammatory and cytosolic noncanonical caspase-11 inflammasome (11–13). LPS is captured by lipopolysaccharide binding protein and transferred to CD14 and then interacts with the TLR4/MD-2 complex. The structural and functional basis of LPS recognition by the TLR4/MD-2 complex is known (14). The binding affinity of LPS for TLR4/MD-2 is greatly influenced by the fatty acid and phosphate groups attached to the glucosamine sugars of lipid A (14, 15). Modifications to lipid A phosphate and/or acyl chain content or length can change LPS from a potent agonist to a nonstimulatory or strong antagonist (16, 17). LPS interaction with TLR4/MD-2 triggers dimerization which initiates downstream signaling cascades, which ultimately result in an inflammatory response critical for pathogen clearance (18). TLR4/MD-2 signaling occurs through myddosome (MyD88)- and triffosome (TRIF)-dependent pathways resulting in secretion of proinflammatory cytokines and type I interferons, respectively (19, 20). MyD88 signaling is enhanced by CD14 at low LPS concentrations; however, at high concentrations activation of the MyD88 pathway can occur in the absence of CD14 (21). CD14-LPS complex endocytosis is not TLR4 dependent; however, TRIF pathway activation occurs only after endocytosis of TLR4/MD-2 dimers, a process reliant on CD14 (21). In comparison to the TLR4/MD-2 signaling pathway, much less is known about the molecular aspects of the LPS noncanonical caspase-11 inflammasome. Intracellular LPS binds directly to caspase-11 CARD motifs through the lipid A moiety, leading to oligomerization of caspase-11 to induce inflammasome activation (22). However, similarly to LPS-TLR4/MD-2 interactions (16), the number of acyl chains on lipid A is important for binding caspase-11 CARD motifs (22).

Here we investigated the inflammatory properties of *C. trachomatis* LPS to determine whether it is capable of functioning as a proinflammatory PAMP. Our results indicate that *C. trachomatis* LPS is nontoxic for mice and a poor inducer of both the canonical TLR4 and noncanonical cytosolic caspase-11 inflammatory pathways in mouse bone marrow-derived macrophages (BMDM). We propose that chlamydiae have evolved a unique lipid A structure with minimal proinflammatory properties as a pathogenic strategy necessary for the establishment of asymptomatic infection.

## RESULTS

### *C. trachomatis* LPS is a poor inducer of endotoxic shock.

**FIG 1:**
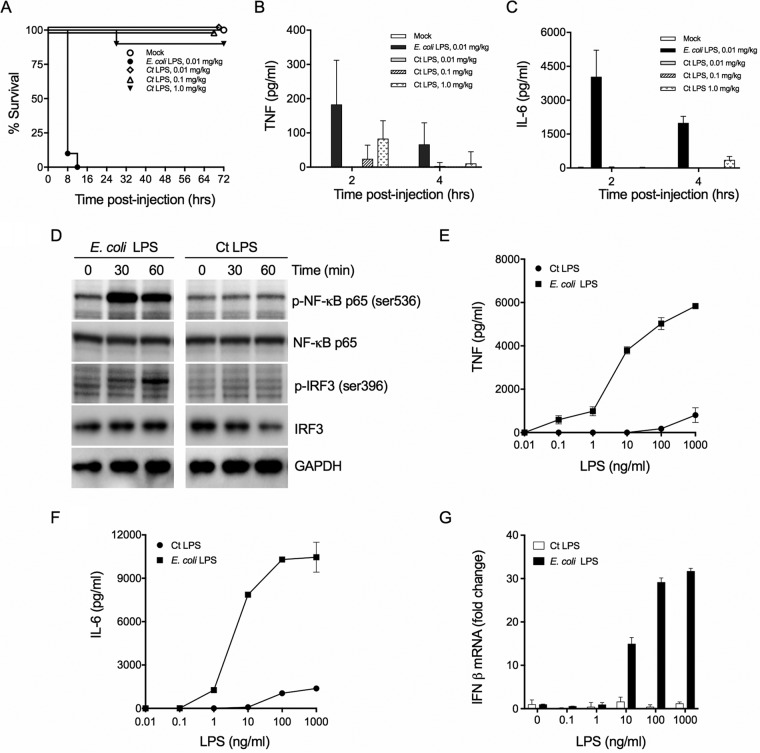
*C. trachomatis* (Ct) LPS does not activate the proinflammatory canonical pathway. (A) Mice (*n* = 5) sensitized with d-GalN were injected i.p. with E. coli or *C. trachomatis* LPS, and survival was monitored at the indicated times. Five mice per group were injected with LPS. Kaplan-Meier curves were generated by Prism 7. (B and C) Sera were collected at 2 and 4 h postinjection and analyzed by ELISA for TNF (B) and IL-6 (C). (D) Western blot analysis of BMDM treated with E. coli or *C. trachomatis* LPS (100 ng/ml) at the indicated times. Lysates prepared from BMDM were resolved on SDS-PAGE gels, transferred to PVDF, and immunoblotted with antibodies against NF-κB p65, phospho-NF-κB p65 (ser536), IRF3, p-IRF3 (ser396), and GAPDH. (E and F) BMDM were treated with various LPS concentrations, and the supernatants were collected and analyzed by ELISA for TNF and IL-6, respectively. (G) BMDM were similarly treated with LPS, and IFN-β mRNA was measured by qRT-PCR. Data shown as mean ± SD from three technical replicates (E, F, and G) and representative of three independent experiments (D, E, F, and G).

Enteric LPS causes endotoxic shock when administered systemically to animals. The endotoxic properties of *C. trachomatis* LPS have not been reported. We therefore tested *C. trachomatis* LPS endotoxicity using the d-galactosamine-sensitized mouse model (23). Endotoxic shock was determined following administration of Escherichia coli LPS and *C. trachomatis* LPS by monitoring mouse morbidity and mortality after LPS injection (Fig. 1A). E. coli LPS was lethal for mice following intraperitoneal (i.p.) injection of 0.01 mg/kg of body weight. In contrast, all mice survived following i.p. injection of *C. trachomatis* LPS at 0.01 and 0.1 mg/kg, and 95% of the mice survived after i.p. injection of 1 mg/kg. *C. trachomatis* LPS exhibited a highly attenuated capacity to induce endotoxin-mediated shock in comparison to E. coli LPS. As expected, elevated levels of TNF and IL-6 were detected in the blood of mice injected with E. coli LPS (Fig. 1B and C). In keeping with the lack of endotoxicity, following injection of even high concentrations of *C. trachomatis* LPS proinflammatory cytokines were not detected in blood or were present at very low levels (Fig. 1B and C).

### *C. trachomatis* LPS does not activate MyD88- or TRIF-dependent pathways.

We next investigated whether *C. trachomatis* LPS was capable of inducing proinflammatory cytokine and type I interferon responses in primary BMDM. CD14-bound LPS is transferred to TLR4/MD-2, promoting dimerization/activation and subsequent intracellular signaling. The TLR4/MD-2 complex signals through two major pathways: (i) the myeloid differentiation primary response protein 88 (MyD88)-dependent pathway with subsequent NK-κB transcriptional signaling of proinflammatory cytokine synthesis and the (ii) TRIF-dependent pathway with subsequent IRF3-dependent IFN-β transcriptional signaling (21). To determine if *C. trachomatis* LPS could stimulate either pathway, immunoblots were probed for NF-κB and IRF3 serine phosphorylation, respectively. BMDM were treated with different concentrations of E. coli or *C. trachomatis* LPS, and serine phosphorylation of NF-κB and IRF3 was monitored by Western blotting (Fig. 1D). In contrast to E. coli LPS, *C. trachomatis* LPS-treated BMDM did not show serine phosphorylation of NF-κB or IRF3. In agreement with this, we found that E. coli, but not *C. trachomatis*, LPS-treated BMDM secreted MyD88 pathway-dependent cytokines TNF and IL-6 (Fig. 1E and F) and produced TRIF-dependent IFN-β mRNA transcripts (Fig. 1G) in a dose-dependent manner. These findings demonstrate that *C. trachomatis* LPS cannot activate the MyD88- or TRIF-dependent pathways, which are consistent with *in vivo* chlamydial genital tract infection findings where TLR4 was not essential for the development of oviduct inflammatory pathology (24).

### *C. trachomatis* LPS binds CD14 and induces CD14 endocytosis.

**FIG 2:**
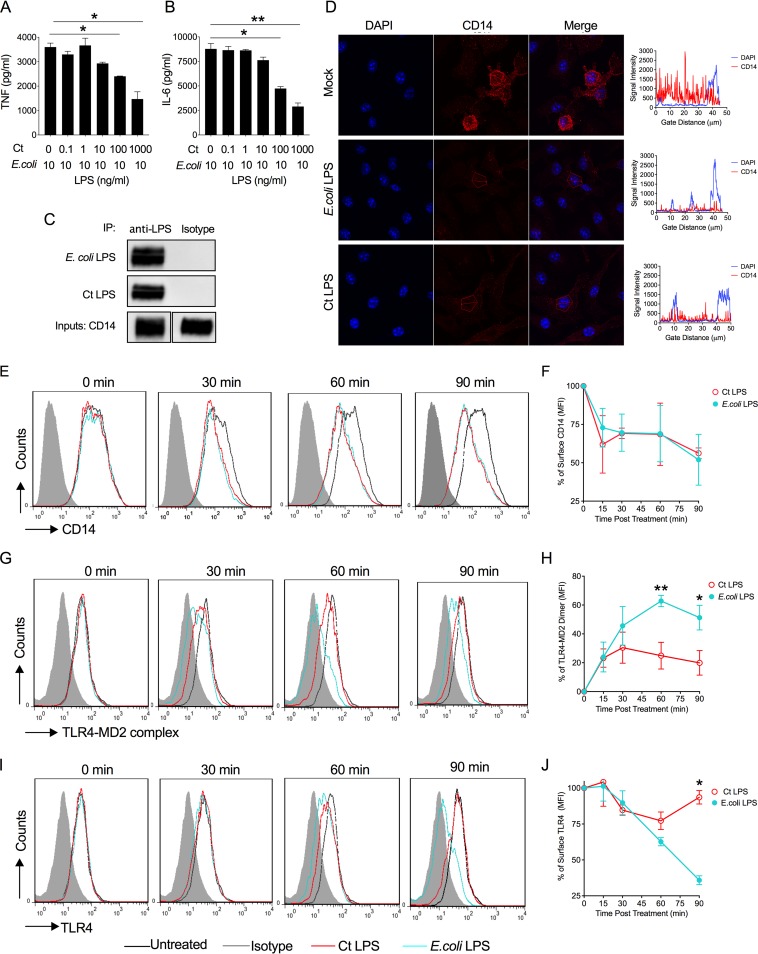
*C. trachomatis* LPS binds CD14 but does not promote dimerization of surface TLR4/MD-2 complex. (A and B) *C. trachomatis* LPS competes in a dose-dependent manner with E. coli LPS. BMDM were treated with a combination of 10 ng/ml E. coli LPS and various increasing concentrations of *C. trachomatis* LPS, and culture supernatants were analyzed by ELISA for TNF (A) and IL-6 (B) cytokine secretion. (C) Coimmunoprecipitation of recombinant CD14-LPS complexes with LPS MAbs. *C. trachomatis* or E. coli LPS was incubated with recombinant mouse CD14 and then immunoprecipitated with Dynabeads magnetic beads conjugated to anti-E. coli LPS or anti-*C. trachomatis* LPS or isotype control (mouse IgG2a) antibodies. Precipitates were subjected to SDS-PAGE, transferred to PVDF, and immunoblotted with anti-CD14 antibody. (D) *C. trachomatis* LPS and *E. coli* LPS both induced endocytosis of CD14 as measured by a reduction in CD14 surface staining. (E) Temporal flow cytometry analysis of surface CD14 expression on BMDM treated with *C. trachomatis* LPS or E. coli LPS. (F) Percentage of surface CD14 staining on *C. trachomatis* LPS- or E. coli LPS-treated BMDM. (G) Temporal flow cytometry analysis of surface TLR/MD-2 complex. BMDM were treated with E. coli LPS and *C. trachomatis* LPS (100 ng/ml) and stained with MAb MTS510, which binds only to monomeric TLR4/MD-2 complex. (H) Percentage of TLR4/MD-2 dimer formation as determined by flow cytometry. *C. trachomatis* LPS induced dimerization of surface TLR4/MD-2 complexes but was significantly (*P* < 0.05) less efficient than E. coli LPS. (I) Temporal flow cytometry analysis of surface TLR4. BMDM were treated with E. coli LPS and *C. trachomatis* LPS (100 ng/ml) and stained with MAb SA15-21, which reacts with total cell surface TLR4. (J) Percentage of TLR4 endocytosis as determined by flow cytometry. *C. trachomatis* LPS induced TLR4 endocytosis but was significantly (*P* < 0.05) less efficient than E. coli LPS. Data shown as mean ± SD from three technical replicates (A, B, F, H, and J) and representative of three independent experiments (A to J). (A, B, H, and J) Two-tailed unpaired Student’s *t* test (*, *P* < 0.05; **, *P* < 0.01).

To better understand the lack of *C. trachomatis* LPS immunostimulatory activity, we performed competitive binding experiments using BMDM treated with a constant amount of E. coli LPS and increasing concentrations of *C. trachomatis* LPS and measuring levels of TNF and IL-6 in the supernatants. *C. trachomatis* LPS effectively antagonized the binding of E. coli LPS in a concentration-dependent manner (Fig. 2A and B). To determine if *C. trachomatis* LPS binds CD14, we performed a coimmunoprecipitation assay using recombinant CD14, LPS, and LPS MAbs. Both E. coli LPS and *C. trachomatis* LPS were bound by recombinant CD14 (Fig. 2C). We next asked whether CD14 binding of *C. trachomatis* LPS was sufficient to promote CD14 endocytosis (21). This was first investigated by immunofluorescence staining of surface CD14 on BMDM treated with LPS (21). *C. trachomatis* LPS and E. coli LPS both induced endocytosis of CD14 as measured by a reduction in CD14 surface staining (Fig. 2D). We confirmed these results using a temporal kinetic flow cytometry analysis measuring CD14 membrane surface expression and receptor recycling (Fig. 2E and F). *C. trachomatis* LPS and E. coli LPS both induced endocytosis of CD14 with similar temporal kinetics.

### *C. trachomatis* LPS does not induce TLR4/MD-2 dimerization or endocytosis.

CD14 transfers LPS to TLR4/MD-2, promoting complex dimerization/activation and initiates subsequent downstream signaling cascades through the MyD88-dependent and TRIF-dependent pathways. The MyD88-dependent pathway signals directly through surface dimerized TLR4/MD-2. In contrast, TRIF signaling requires CD14-dependent endocytosis of the TLR4/MD-2 complex (18). To determine if *C. trachomatis* LPS was capable of promoting TLR/MD-2 dimerization and CD14-dependent TLR4/MD-2 endocytosis, we used temporal kinetic flow cytometry analysis measuring TLR4/MD-2 membrane surface expression and endocytosis (25). TLR4/MD-2 dimerization was investigated by using MAb MTS510, which recognizes TLR/MD-2 monomers only at the plasma membrane; a loss of surface staining of MTS510 represents TLR4/MD-2 dimerization induced by LPS (25, 26). As shown in Fig. 2G and H, E. coli LPS treatment results in efficient temporally dependent dimerization of surface TLR4/MD-2 complex. In contrast, *C. trachomatis* LPS induced significantly less dimerization of surface TLR/MD-2 complexes (Fig. 2G and H). TLR4/MD-2 endocytosis was studied by using the MAb SA15-21, which recognizes total surface TLR4. The reduction of total surface TLR4 indirectly represents TLR4 endocytosis (25). Surface staining of total TLR4 of BMDM treated with E. coli and *C. trachomatis* LPS, respectively, at 30, 60, and 90 minutes posttreatment is shown in Fig. 2I and J. E. coli LPS treatment results in a marked time-dependent reduction of total surface TLR4. In contrast, *C. trachomatis* LPS induced significantly less TLR4 endocytosis (Fig. 2I and J). Collectively, our results support the conclusion that while binding efficiently to CD14, *C. trachomatis* LPS cannot effectively induce TLR4/MD-2 complex dimerization or endocytosis, which provides a molecular mechanism for the inability of *C. trachomatis* LPS to activate both the MyD88- and TRIF-dependent pathways.

### *C. trachomatis* LPS does not activate the noncanonical inflammasome pathway.

**FIG 3:**
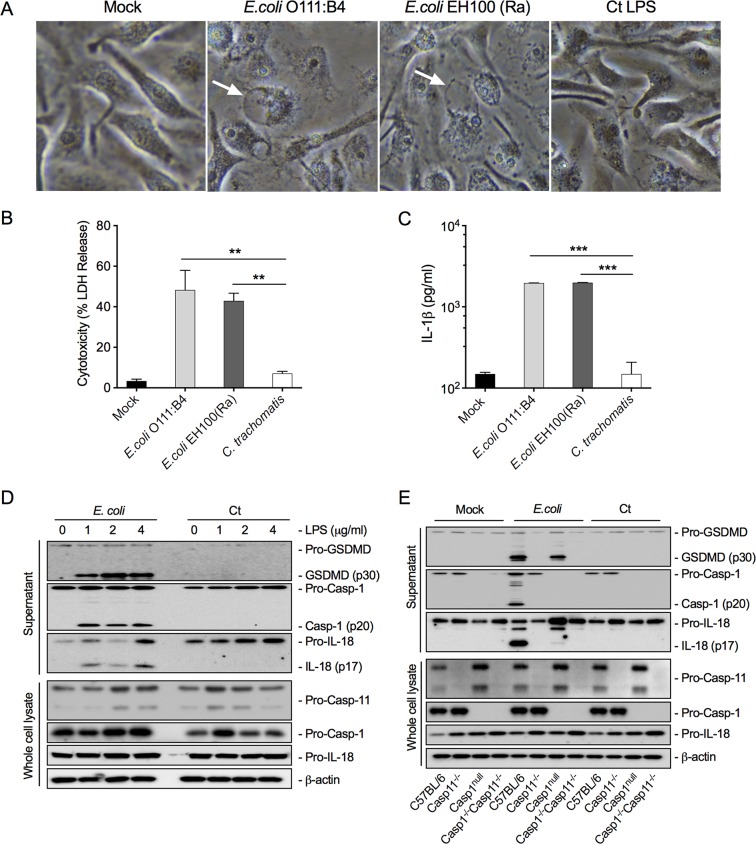
*C. trachomatis* LPS does not activate the noncanonical caspase-11 inflammasome pathway. (A) BMDM transfected with *C. trachomatis* LPS does not exhibit a pyroptotic cell ballooning morphology or cause BMDM cytotoxicity. Phase photomicrographs (40×) of BMDM mock treated or treated with E. coli (O111:B4) smooth LPS, E. coli (EH100) rough LPS, or *C. trachomatis* LPS. (B and C) Similarly treated BMDM assayed for cytotoxicity as measured by LDH release (B) or IL-1β secretion (C). (D) BMDM treated with E. coli or *C. trachomatis* LPS at different LPS concentrations were assayed by immunoblotting for cleavage of GSDMD, caspase-1, and IL-18 in BMDM supernatants. Pro-caspase-11, pro-caspase-1, and pro-IL-18 were assayed by immunoblotting in BMDM cell lysates. (E) Confirmation of E. coli LPS-specific activation of caspase-11 using BMDM from C57BL/6 caspase-1^−/−^ and caspase-11^−/−^ mice and caspase-1^−/−^ caspase-11^−/−^ double KO mice. Cytosolic E. coli LPS specifically activates caspase-11 in C57BL/6 and caspase-1^−/−^ BMDM. Note that cytosolic E. coli but not *C. trachomatis* LPS activated the caspase-11 as shown by proteolytic cleavage of GSDMD, caspase-1, and IL-18. Data shown as mean ± SD from three technical replicates (B and C) and representative of three independent experiments (B, C, D, and E). (B and C) Two-tailed unpaired Student’s *t* test (**, *P* < 0.01; ***, *P* < 0.001).

It is known that the PAMP receptor for cytosolic LPS is caspase-11 (human orthologs are caspase-4 and -5) and that engagement of caspase-11 is a key factor in activating the noncanonical inflammasome pathway (12, 13). Chlamydia growth is confined to the inclusion. In order for chlamydial LPS to target caspase-11 activation, it must gain access to the host cytoplasm. In this regard, it has been shown that interferon-induced GTPases promote inclusion ubiquitination resulting in premature inclusion lysis, providing a mechanism for LPS access to the cytosol (27, 28). To determine if cytosolic *C. trachomatis* LPS could activate caspase-11, we transfected BMDM with chlamydial LPS. A phenotypic characteristic of cells transfected with E. coli LPS is the formation of a “ballooning” morphology associated with pyroptosis and cytotoxic cell death as measured by LDH release (29). Transfection of BMDM with E. coli LPS, but not *C. trachomatis* LPS, resulted in a pyroptotic ballooning morphology (Fig. 3A) and characteristic cytotoxicity assayed by LDH release and IL-1β production (Fig. 3B and C). We tested both smooth (E. coli O111:B4) and rough (EH100-Ra) E. coli LPS in these experiments as the *C. trachomatis* LPS structure is more similar to that of EH100-Ra. Both types of E. coli LPS, but not chlamydial LPS, caused cell ballooning and pyroptotic cell death.

LPS-activated caspase-11 promotes the cleavage of gasdermin D (GSDMD) into its cytoplasmic membrane pore, forming an N-terminal p30 fragment causing osmotic cell death associated with pyroptosis (13). Additionally, caspase-11 binding of enteric LPS activates pro-caspase-1 through NLRP3 and ASC to produce active caspase-1. Active caspase-1 (p20) then cleaves pro-IL-1β and pro-IL-18 to mature active inflammatory IL-1β and IL-18 polypeptides that are secreted through GSDMD cytoplasmic pores (30). We performed Western blot analysis on E. coli and *C. trachomatis* LPS-transfected BMDM to determine whether the noncanonical inflammasome pathway was activated by *C. trachomatis* LPS. Results from the blot analyses showed that E. coli LPS induced cleavage of GSDMD to its active p30 fragment, pro-caspase-1 to its active p20 fragment, and pro-IL-18 to its active p17 fragment (Fig. 3D). Notably, cytosolic *C. trachomatis* LPS did not result in the cleavage of GSDMD, caspase-1, or IL-1β.

To corroborate and expand on these findings, BMDM derived from B6 knockout (KO) mice with genetic deficiencies in caspase-1 or -11 or both were used to study E. coli and *C. trachomatis* LPS interactions with the caspase-11 pathway. Wild-type, caspase-11^−/−^, caspase-1^null^, and caspase-1^−/−^/11^−/−^ BMDM were transfected with E. coli or *C. trachomatis* LPS and analyzed by Western blotting using antibodies specific to pro- and cleaved forms of GSDMD, caspase-1, IL-1, and IL-18. As expected, cytosolic E. coli LPS resulted in the cleavage of GSDMD, caspase-1, IL-1, and IL-18 in WT and caspase-1^null^ BMDM but did not result in cleavage of these targets in caspase-11^−/−^ or caspase-1^−/^^−^/11^−/−^ mice. Conversely, cytosolic *C. trachomatis* LPS failed to signal through caspase-11 as shown by the lack of GSDMD, caspase-1, and IL-18 cleavage in WT, caspase-11^−/−^, caspase-1^null^, and caspase-1^−/−^/11^−/−^ BMDM (Fig. 3E). We conclude that *C. trachomatis* LPS is not a cytosolic PAMP for activating the caspase-11-dependent noncanonical inflammasome pathway. In total, our results support the conclusion that *C. trachomatis* LPS does not function as a PAMP in activating either the canonical or noncanonical inflammatory pathways.

## DISCUSSION

A characteristic feature of C. trachomatis urogenital infection is the high incidence of asymptomatic infections. This is unusual for Gram-negative organisms that have LPS, the prototypical proinflammatory PAMP. Previous studies have shown that chlamydial LPS is less toxic than enteric LPS, although the molecular basis for this has remained undefined (8, 9).

**FIG 4:**
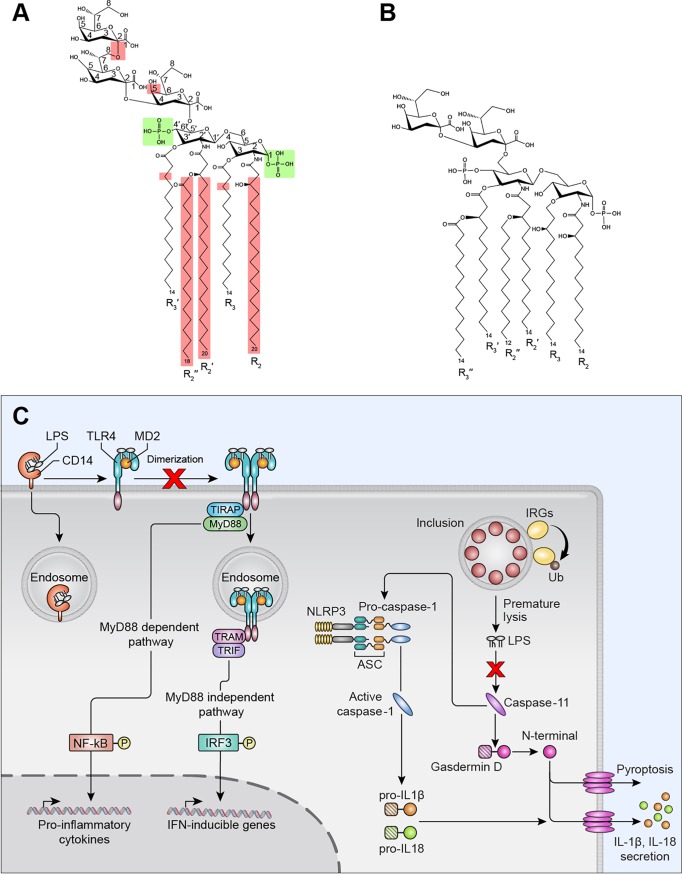
Structure and functional relationship of *C. trachomatis* LPS and subversion of canonical and noncanonical inflammatory pathways. (A and B) Structures of chlamydial and E. coli LPS, respectively. *C. trachomatis* LPS shares the same diphosphorylated glucosamine as E. coli LPS, highlighted in green, an essential structural feature for CD14 binding (25). Structural characteristics of *C. trachomatis* LPS that differ from E. coli LPS are highlighted in red. (1) A 2-8, 2-4-linked tri-Kdo and the lack of LPS heptosyl transferase 1 (*waaC*) in the chlamydial genome prevent further glycosylation; thus, chlamydial LPS has a deep rough Re phenotype that avoids PRR recognition. (2) Myristoylated instead of (*R*)-3-hydroxymyristoyl chains at positions R3′ of Kdo3-lipid IVA, thereby preventing further acylation. (3) Chlamydial LPS amide-linked acyl and 3′ oxyacyl groups (R2, R2′, and R2″) are unusually long, C_18–20_ compared to C_12–14_. (C) A model of *C. trachomatis* LPS subversion of canonical and noncanonical inflammatory pathways.

The fact that infection remains silent suggests that chlamydial LPS is a poor agonist of the canonical and noncanonical inflammatory pathways. Collectively, our findings show that *in vivo* chlamydial LPS is nontoxic and *in vitro* displays limited activation of PRRs that function in the proinflammatory canonical TLR4 and noncanonical caspase-11 inflammasome pathways. Therefore, chlamydial LPS is an immunologically silent molecule that fails to trigger inflammatory innate immunity, which may in part explain the high incidence of C. trachomatis asymptomatic infections. Since asymptomatic infection in women can ascend and cause chronic inflammatory disease such as PID, the findings reported here would not support a role for *C. trachomatis* LPS in driving upper genital tract immunopathology through either the canonical TLR4 or the noncanonical caspase-1/11 pathways. We conclude that these broad innate immunity silencing characteristics are the result of chlamydia’s unique LPS structure (Fig. 4).

A vast array of lipid A modification systems exists in Gram-negative pathogens. The resulting lipid A structures often show reduced immunostimulatory effects, suggesting that lipid A modification is an important immune evasion strategy. Lipid A modification requires addition of new enzymes, which comes at a cost of increased genome size (16, 17). Chlamydia’s lifestyle as an obligate intracellular pathogen has introduced strong selective pressure to maintain a minimally sized genome (31, 32). Therefore, chlamydiae have evolved a strategy to alter their lipid A structure that reduces its toxicity without the need for additional genetic coding capacity. To this point, we show that chlamydial LPS binds to and induces CD14 endocytosis, is a weak inducer of TLR4/MD-2 dimerization, and inefficiently promotes TLR4 endocytosis. Chlamydial LPS binds CD14, and the resulting complex is endocytosed, a property consistent with it having two phosphates (1 and 4′) attached to lipid A glucosamine sugars, which are an essential structural feature for CD14 endocytosis (25). Rhodobacter sphaeroides LPS displays similar structural and functional characteristics as chlamydial LPS. R. sphaeroides LPS is penta-acylated, is diphosphorylated, binds CD14, and promotes efficient endocytosis but is a poor inducer of TLR4 dimerization and subsequent endocytosis (25). The structural features of chlamydial LPS resulted in a CD14 deficiency at the plasma membrane (Fig. 2D to F). The ability of chlamydial LPS to provoke a CD14 deficiency at the cell surface is consistent with its ability to function as an effective TLR4 antagonist of E. coli LPS (Fig. 2A and B). From a pathogenesis standpoint, a reduction in CD14 surface expression could dampen the proinflammatory response to Gram-negative pathogens, such as Neisseria gonorrhoeae, a common chlamydial coinfecting pathogen of the urogenital tract. How such host-pathogen-microbiome interactions might affect chlamydial growth or natural host resistance to coinfecting pathogens of the urogenital tract remains to be determined.

Structurally, chlamydial lipid A is penta-acylated, not hexa-acylated (Fig. 4). Numerous functional and structural studies have led to the conclusion that LPS variants with fewer than six acyl chains are poor inducers of the TLR4 pathway (16, 17). Lack of chlamydial LPS-induced TLR/MD-2 dimerization, which is essential for myddosome signaling (18, 25), is sufficient to explain the limited production of the proinflammatory cytokines IL-6 and TNF (Fig. 1D and E). Likewise, the lack of chlamydial LPS-induced TLR4 endocytosis, which is required for triffosome signaling (18, 25), offers a molecular explanation for limited IFN-β mRNA production (Fig. 1F). Together, these findings are consistent with the observation that chlamydial LPS displayed essentially no endotoxic activity *in vivo*. Chlamydiae have evolved two features which ensure that penta-acylated lipid A cannot be converted to the more inflammatory hexa-acylated form. First, chlamydiae lack *lpxM*, encoding an acyltransferase that catalyzes the secondary acylation of the R-3-hydroxyacyl chain at position 3′ of Kdo_3_-lipid IV_A_ (Fig. 4A) (31). Second, the lipid A of chlamydial LPS is unusual in that it contains myristoylated instead of (*R*)-3-hydroxymyristoyl chains at positions R3 and R3′ (Fig. 4A) (8, 33). This structural feature is a direct consequence of chlamydial UDP-*N*-acetylglucosamine acyltransferase (*C. trachomatis* LpxA) having a strong preference for a nonhydroxylated acyl-acyl carrier protein (ACP) to a hydroxyacyl-ACP (34). Chlamydiae therefore cannot generate a 3′-acyloxyacyl unit in the sixth acyl chain of lipid A.

Chlamydial LPS amide-linked acyl and 3′-oxyacyl groups (R2, R2′, and R2″, Fig. 4A) are C_18–20_ in length, compared to C_12–14_ in E. coli (Fig. 4B). Alterations in acyl chain length and degree of saturation have a profound impact on immune activation characteristics (16, 17). Chlamydiae encode the capacity to synthesize branched-chain fatty acids *de novo* (31, 35) and have the capability to salvage straight-chain fatty acids from their host (36). In marked contrast to the acyl chains of chlamydial glycerophospholipids, which are primarily branched chains (37), the LPS lipid A acyl chains are predominately straight chains (8). This dichotomy raises the intriguing possibility that chlamydiae salvage host fatty acids for lipid A synthesis as a pathogenic strategy, resulting in an LPS structure that is a poor TLR4 or caspase-11 agonist. To determine whether conversion of penta-acylated lipid A to hexa-acylated lipid A would increase chlamydial LPS toxicity would require genetic deletion of *C. trachomatis lpxA*, replacing it with E. coli *lpxA* and adding E. coli *lpxM*. While systems for genetically manipulating chlamydiae are now available (38), the more complex genetic manipulations required for these studies require further development.

A crystal structure model of enteric LPS–TLR4/MD-2 interactions indicates that five of the six lipid chains of LPS are buried deep inside the large hydrophobic pocket of MD-2 (15, 39). This interaction promotes TLR4/MD-2 dimerization required for downstream signal transduction pathways that produce serine phosphorylation of NF-κB and IRF3, leading to the transcription of proinflammatory cytokines and type I interferons (18). The combination of penta-acylation and long C_18–20_ acyl chains likely affects the way that chlamydial LPS binds the hydrophobic MD-2 pocket, thus preventing the conformational changes required for efficient TLR4 dimerization and activation of myddosome and triffosome signaling (15, 18).

Our morphological, microscopic, and Western blotting results clearly show that chlamydial LPS does not activate the noncanonical inflammasome pathway (Fig. 3). In contrast to hexa-acylated E. coli LPS, which strongly activates the noncanonical inflammasome pathway, R. sphaeroides LPS, which is penta-acylated and tetra-acylated, lipid IVA binds caspase-11 but fails to promote its oligomerization and activation (13, 22). We conclude that the inability of chlamydial LPS to activate the noncanonical inflammasome is also likely a result of its hypoacylated lipid A structure (8).

## MATERIALS AND METHODS

### Lipopolysaccharide.

E. coli O111:B4 LPS was purchased from Sigma-Aldrich (catalog no. L4391; St. Louis, MO), E. coli EH100 (Ra) LPS (catalog no. HC4046) was from Hycult Biotech Inc. (Plymouth, PA); C. trachomatis serovar E LPS has been analyzed structurally by mass spectrometry (8) and was purchased from Glycobiotech GmbH (Kükels, Germany). Lyophilized LPS was resuspended in Ca^2+^- and Mg^2+^-free Hanks balanced salt solution (HBSS) or triethylamine at 1 mg/ml and stored at −20°C. LPS serotypes were verified utilizing the Pro-Q Emerald 300 lipopolysaccharide gel stain kit (Molecular Probes Inc., Eugene, OR).

### Mice.

Six- to 8-week-old C57BL/6J, caspase 11^−/−^ and caspase-1^−/−^ caspase-11^−/−^ mice were purchased from Jackson Laboratories. Caspase-1^null^ mice were provided by Thirumala-Devi Kanneganti, St. Jude Children’s Research Hospital (40). All procedures were performed in accordance with the guidelines of the National Institutes of Health Institutional Animal Care and Use Committee.

### d-Galactosamine-sensitized LPS challenge.

Female C57BL/6J mice were injected intraperitoneally (i.p.) with E. coli O111:B4 LPS (0.01 mg/kg) or different doses of *C. trachomatis* LPS (0.01, 0.1, or 1.0 mg/kg) in combination with d-galactosamine (800 mg/kg). Mice were observed for moribundity and lethality over a 72-h period.

### BMDM cultures.

Briefly, bone marrow cells were cultured in MEM–F-12 supplemented with 0.1 IU/ml M-CSF, 10% heat-inactivated fetal bovine serum (HyClone), 100 IU/penicillin, 100 µg/ml streptomycin, and 10 mM l-glutamine. Cultures were maintained in 5% CO_2_ at 37°C in non-tissue-culture-coated petri dishes (Corning Inc.). On day 3, supernatants were gently removed and replaced with the same volume of culture medium. The nonadherent cells were removed on day 7, and adherent BMDM were dislodged with Cellstripper nonenzymatic cell dissociation solution (Thermo Fisher Scientific), pooled, and seeded into 96- or 24-well plates (Corning). BMDM seeded into 24-well plates were incubated with different concentrations of E. coli LPS or *C. trachomatis* LPS. Culture supernatants were assayed by ELISA for TNF and IL-6 secretion, and adherent BMDM were solubilized in Laemmli sample buffer and used for immunoblotting. For transfection experiments, BMDM seeded into 96-well plates overnight were primed for 6 h with 1 μg/ml Pam3CSK4 in serum-free Opti-MEM (Life Technologies). Primed cells were transfected with 1, 2, or 4 μg/ml E. coli or *C. trachomatis* LPS by using 0.25% (vol/vol) FuGENE HD transfection reagent (Promega) or mock transfected with FuGene HD for 12 h as previously described (12). Supernatants were collected and assayed by immunoblotting; LDH cytotoxicity or IL-1β secretion was assayed by ELISA.

### Flow cytometry.

We used the flow cytometry procedure described by Kagan et al. to monitor TLR4 endocytosis and TLR4-MD2 dimerization (41). BMDM were treated with 200 ng/ml *C. trachomatis* LPS or E. coli O111:B4 LPS suspended in DMEM supplemented with 10% FBS and incubated prior to flow cytometry analysis. Cells were stained with appropriate fluorochrome-labeled specific antibodies or isotype control antibodies. Mouse antibodies used were anti-CD11b (clone M1/70; BioLegend), anti-CD14 (clone Sa14-2; BioLegend), anti-TLR4 (clone SA15-21; BioLegend), and anti-TLR4/MD-2 complex (clone MTS-510; Thermo Fisher Scientific). Cells were processed with the Becton, Dickinson (BD) Fortessa flow cytometer using FACSDiva software (BD). Flow cytometry experiments were analyzed using FlowJo (Tree Star Inc.).

### ELISA.

Mouse IL-6 and TNF ELISA sets were purchased from BD Biosciences (Franklin Lakes, NJ). Cell culture supernatants and mouse sera were assayed according to the manufacturer’s protocols. ELISA was performed in two independent experiments, each done in triplicate.

### Quantitative RT-PCR.

Total RNA from E. coli or *C. trachomatis* LPS-treated BMDM was isolated using TRIzol reagent (Invitrogen) and the RNeasy RNA extraction kit according to the manufacturer’s instructions. The level of IFN-β mRNA was determined by using the SuperScript III Platinum SYBR Green one-step qRT-PCR kit (Invitrogen) according to the manufacturer’s instructions. Primers for murine IFN-β and GAPDH were purchased from Qiagen. Samples were analyzed on a 7500 Fast Real-Time PCR system (Applied Biosystems). All experiments were performed in triplicate.

### Immunoblotting.

BMDM were seeded in 24-well plates and treated with E. coli or *C. trachomatis* LPS. Cells were lysed with RIPA buffer. Proteins in culture supernatants were precipitated with 7.2% trichloroacetic acid plus 0.15% sodium cholate and were resuspended with NuPAGE LDS sample buffer (12). Samples were subjected to SDS-PAGE and transferred to either PVDF or nitrocellulose for immunoblotting, respectively. Blots were blocked overnight at 4°C in PBS with 0.1% Tween 20 and 5% nonfat dry milk. Primary antibodies used include WN1 222-5 (Hycult Biotech), NF-κB (clone L8F6; Cell Signal), pNF-κB (clone 93H1; Cell Signal), IRF3 (clone D83B9; Cell Signal), pIRF3Ser396 (clone 4D4G; Cell Signal), GAPDH (clone 14C10; Cell Signal), pro-GSDMD (clone A-7; Santa Cruz), GSDMD (p30) (Abcam), caspase-1 (p20) (clone Casper-1; AdipoGen), pro-IL-18 (Biovison), IL-18 (Biovison), pro-caspase-11 (clone 17D9; Sigma), and beta-actin (Sigma). Horseradish peroxidase-conjugated secondary antibodies were used for detection (Invitrogen).

### Cell cytotoxicity assay.

Supernatants from BMDM transfected with E. coli and *C. trachomatis* LPS were collected, and LDH was measured by using the CytoTox 96 nonradioactive cytotoxicity assay (Promega) according to the manufacturer’s instructions. All values represent the percentage of LDH released into supernatants compared to a maximum lysis control.

### Coimmunoprecipitation.

One microgram of recombinant mouse CD14 (Sino Biological) was incubated with 1 µg of *C. trachomatis* or E. coli EH100 (Ra) LPS in 10 mM Tris-HCl (pH 7.5)–0.15 M NaCl and incubated at 37°C for 1 h. Dynabeads magnetic beads (Invitrogen) coupled to *C. trachomatis* and E. coli LPS-specific antibodies (MAbs EVIH1 and WN1 222-5, respectively) or appropriate isotype control (IgG2b) were added according to manufacturer’s specifications and incubated for 30 min at room temperature. Samples were then washed with PBS (pH 7.4) three times before elution into Laemmli buffer for SDS-PAGE. Immunoblots were probed with mouse anti-CD14 and HRP-conjugated goat anti-mouse secondary antibody.

### Immunofluorescence.

BMDM (2 × 10^5^) were plated on glass coverslips in 24-well plates and treated with 1 μg/ml of *C. trachomatis* LPS or 1 μg/ml E. coli LPS and or were untreated as a control. At 5 min posttreatment, cells were fixed in 2% paraformaldehyde (PFA) for 10 min at room temperature and blocked for 1 h in a 1× PBS solution containing 0.3% Triton X-100 and 100 mg/ml goat serum at room temperature. Cells were immunostained with 1× PBS-1% BSA-0.3% Triton solution containing anti-CD14 antibody. Primary antibody rat anti-mouse CD14 (4C1) was used at a 1:200 dilution. Secondary antibodies were used at 1:400 (Alexa Fluor 555 goat anti-rat). Coverslips were stained with DAPI at 1:1,000 in PBS for 5 min and mounted using Prolong Gold. High-resolution images were captured using a Zeiss 880 laser scanning microscope with an Airyscan detector. Intensity histograms measuring signal intensity of blue-channel DAPI and red-channel anti-CD14 were produced by tracing a histogram line through representative cell populations and processed in Zen Black (Carl Zeiss Imaging). Z-stack projections were imaged at an interval of 0.2 μm. All images were processed in Zen Blue and Zen Black (Carl Zeiss Imaging).

### Statistical analysis.

The statistical significance of differences between data groups was determined by the unpaired Student *t* test using GraphPad Prism 7.

## References

[1] Wiesenfeld HC, Sweet RL, Ness RB, Krohn MA, Amortegui AJ, Hillier SL 2005 Comparison of acute and subclinical pelvic inflammatory disease. Sex Transm Dis 32:400–405. doi:10.1097/01.olq.0000154508.26532.6a. 15976596

[2] Wiesenfeld HC, Hillier SL, Meyn LA, Amortegui AJ, Sweet RL 2012 Subclinical pelvic inflammatory disease and infertility. Obstet Gynecol 120:37–43. doi:10.1097/AOG.0b013e31825a6bc9. 22678036

[3] Moulder JW 1991 Interaction of chlamydiae and host cells in vitro. Microbiol Rev 55:143–190. 2030670 10.1128/mr.55.1.143-190.1991 PMC372804

[4] Caldwell HD, Hitchcock PJ 1984 Monoclonal antibody against a genus-specific antigen of *Chlamydia* species: location of the epitope on chlamydial lipopolysaccharide. Infect Immun 44:306–314. 6425219 10.1128/iai.44.2.306-314.1984 PMC263518

[5] Rund S, Lindner B, Brade H, Holst O 1999 Structural analysis of the lipopolysaccharide from *Chlamydia trachomatis* serotype L2. J Biol Chem 274:16819–16824. doi:10.1074/jbc.274.24.16819. 10358025

[6] Nano FE, Caldwell HD 1985 Expression of the chlamydial genus-specific lipopolysaccharide epitope in *Escherichia coli*. Science 228:742–744. doi:10.1126/science.2581315. 2581315

[7] Nguyen BD, Cunningham D, Liang X, Chen X, Toone EJ, Raetz CR, Zhou P, Valdivia RH 2011 Lipooligosaccharide is required for the generation of infectious elementary bodies in *Chlamydia trachomatis*. Proc Natl Acad Sci U S A 108:10284–10289. doi:10.1073/pnas.1107478108. 21628561 PMC3121853

[8] Heine H, Muller-Loennies S, Brade L, Lindner B, Brade H 2003 Endotoxic activity and chemical structure of lipopolysaccharides from *Chlamydia trachomatis* serotypes E and L2 and *Chlamydophila psittaci* 6BC. Eur J Biochem 270:440–450. doi:10.1046/j.1432-1033.2003.03392.x. 12542694

[9] Ingalls RR, Rice PA, Qureshi N, Takayama K, Lin JS, Golenbock DT 1995 The inflammatory cytokine response to *Chlamydia trachomatis* infection is endotoxin mediated. Infect Immun 63:3125–3130. 7542638 10.1128/iai.63.8.3125-3130.1995 PMC173426

[10] Heine H, Gronow S, Zamyatina A, Kosma P, Brade H 2007 Investigation on the agonistic and antagonistic biological activities of synthetic *Chlamydia* lipid A and its use in in vitro enzymatic assays. J Endotoxin Res 13:126–132. doi:10.1177/0968051907079122. 17621554

[11] Akira S, Takeda K 2004 Toll-like receptor signalling. Nat Rev Immunol 4:499–511. doi:10.1038/nri1391. 15229469

[12] Kayagaki N, Warming S, Lamkanfi M, Vande Walle L, Louie S, Dong J, Newton K, Qu Y, Liu J, Heldens S, Zhang J, Lee WP, Roose-Girma M, Dixit VM 2011 Non-canonical inflammasome activation targets caspase-11. Nature 479:117–121. doi:10.1038/nature10558. 22002608

[13] Hagar JA, Powell DA, Aachoui Y, Ernst RK, Miao EA 2013 Cytoplasmic LPS activates caspase-11: implications in TLR4-independent endotoxic shock. Science 341:1250–1253. doi:10.1126/science.1240988. 24031018 PMC3931427

[14] Kim HM, Park BS, Kim JI, Kim SE, Lee J, Oh SC, Enkhbayar P, Matsushima N, Lee H, Yoo OJ, Lee JO 2007 Crystal structure of the TLR4-MD-2 complex with bound endotoxin antagonist Eritoran. Cell 130:906–917. doi:10.1016/j.cell.2007.08.002. 17803912

[15] Park BS, Song DH, Kim HM, Choi BS, Lee H, Lee JO 2009 The structural basis of lipopolysaccharide recognition by the TLR4-MD-2 complex. Nature 458:1191–1195. doi:10.1038/nature07830. 19252480

[16] Needham BD, Trent MS 2013 Fortifying the barrier: the impact of lipid A remodelling on bacterial pathogenesis. Nat Rev Microbiol 11:467–481. doi:10.1038/nrmicro3047. 23748343 PMC6913092

[17] Xiao X, Sankaranarayanan K, Khosla C 2017 Biosynthesis and structure-activity relationships of the lipid a family of glycolipids. Curr Opin Chem Biol 40:127–137. doi:10.1016/j.cbpa.2017.07.008. 28942130 PMC5696077

[18] Rosadini CV, Kagan JC 2017 Early innate immune responses to bacterial LPS. Curr Opin Immunol 44:14–19. doi:10.1016/j.coi.2016.10.005. 27842237 PMC5426986

[19] Kagan JC 2017 Lipopolysaccharide detection across the kingdoms of life. Trends Immunol 38:696–704. doi:10.1016/j.it.2017.05.001. 28551077 PMC5624813

[20] Kieser KJ, Kagan JC 2017 Multi-receptor detection of individual bacterial products by the innate immune system. Nat Rev Immunol 17:376–390. doi:10.1038/nri.2017.25. 28461704 PMC6698371

[21] Zanoni I, Ostuni R, Marek LR, Barresi S, Barbalat R, Barton GM, Granucci F, Kagan JC 2011 CD14 controls the LPS-induced endocytosis of Toll-like receptor 4. Cell 147:868–880. doi:10.1016/j.cell.2011.09.051. 22078883 PMC3217211

[22] Yi YS 2017 Caspase-11 non-canonical inflammasome: a critical sensor of intracellular lipopolysaccharide in macrophage-mediated inflammatory responses. Immunology 152:207–217. doi:10.1111/imm.12787. 28695629 PMC5588777

[23] Galanos C, Freudenberg MA, Reutter W 1979 Galactosamine-induced sensitization to the lethal effects of endotoxin. Proc Natl Acad Sci U S A 76:5939–5943. doi:10.1073/pnas.76.11.5939. 293694 PMC411768

[24] Darville T, O'Neill JM, Andrews CW Jr, Nagarajan UM, Stahl L, Ojcius DM 2003 Toll-like receptor-2, but not Toll-like receptor-4, is essential for development of oviduct pathology in chlamydial genital tract infection. J Immunol 171:6187–6197. doi:10.4049/jimmunol.171.11.6187. 14634135

[25] Tan Y, Zanoni I, Cullen TW, Goodman AL, Kagan JC 2015 Mechanisms of Toll-like receptor 4 endocytosis reveal a common immune-evasion strategy used by pathogenic and commensal bacteria. Immunity 43:909–922. doi:10.1016/j.immuni.2015.10.008. 26546281 PMC4685471

[26] Akashi S, Shimazu R, Ogata H, Nagai Y, Takeda K, Kimoto M, Miyake K 2000 Cutting edge: cell surface expression and lipopolysaccharide signaling via the toll-like receptor 4-MD-2 complex on mouse peritoneal macrophages. J Immunol 164:3471–3475. doi:10.4049/jimmunol.164.7.3471. 10725698

[27] Campbell S, Richmond SJ, Yates PS, Storey CC 1994 Lipopolysaccharide in cells infected by *Chlamydia trachomatis*. Microbiology 140:1995–2002. doi:10.1099/13500872-140-8-1995. 7921250

[28] Finethy R, Coers J 2016 Sensing the enemy, containing the threat: cell-autonomous immunity to *Chlamydia trachomatis*. FEMS Microbiol Rev 40:875–893. doi:10.1093/femsre/fuw027. 28201690 PMC5975928

[29] Shi J, Zhao Y, Wang Y, Gao W, Ding J, Li P, Hu L, Shao F 2014 Inflammatory caspases are innate immune receptors for intracellular LPS. Nature 514:187–192. doi:10.1038/nature13683. 25119034

[30] Man SM, Karki R, Kanneganti TD 2017 Molecular mechanisms and functions of pyroptosis, inflammatory caspases and inflammasomes in infectious diseases. Immunol Rev 277:61–75. doi:10.1111/imr.12534. 28462526 PMC5416822

[31] Stephens RS, Kalman S, Lammel C, Fan J, Marathe R, Aravind L, Mitchell W, Olinger L, Tatusov RL, Zhao Q, Koonin EV, Davis RW 1998 Genome sequence of an obligate intracellular pathogen of humans: *Chlamydia trachomatis*. Science 282:754–759. doi:10.1126/science.282.5389.754. 9784136

[32] Hadfield J, Benard A, Domman D, Thomson N 2018 The hidden genomics of *Chlamydia trachomatis*. Curr Top Microbiol Immunol 412:107–131. doi:10.1007/82_2017_39. 29071471

[33] Kosma P 1999 Chlamydial lipopolysaccharide. Biochim Biophys Acta 1455:387–402. doi:10.1016/S0925-4439(99)00061-7. 10571027

[34] Sweet CR, Lin S, Cotter RJ, Raetz CR 2001 A *Chlamydia trachomatis* UDP-N-acetylglucosamine acyltransferase selective for myristoyl-acyl carrier protein. Expression in Escherichia coli and formation of hybrid lipid A species. J Biol Chem 276:19565–19574. doi:10.1074/jbc.M101868200. 11279221

[35] Yao J, Rock CO 2018 Therapeutic targets in chlamydial fatty acid and phospholipid synthesis. Front Microbiol 9:2291. doi:10.3389/fmicb.2018.02291. 30319589 PMC6167442

[36] Yao J, Dodson VJ, Frank MW, Rock CO 2015 *Chlamydia trachomatis* scavenges host fatty acids for phospholipid synthesis via an acyl-acyl carrier protein synthetase. J Biol Chem 290:22163–22173. doi:10.1074/jbc.M115.671008. 26195634 PMC4571967

[37] Yao J, Cherian PT, Frank MW, Rock CO 2015 *Chlamydia trachomatis* relies on autonomous phospholipid synthesis for membrane biogenesis. J Biol Chem 290:18874–18888. doi:10.1074/jbc.M115.657148. 25995447 PMC4521007

[38] Sixt BS, Valdivia RH 2016 Molecular genetic analysis of *Chlamydia* species. Annu Rev Microbiol 70:179–198. doi:10.1146/annurev-micro-102215-095539. 27607551

[39] Ryu JK, Kim SJ, Rah SH, Kang JI, Jung HE, Lee D, Lee HK, Lee JO, Park BS, Yoon TY, Kim HM 2017 Reconstruction of LPS transfer cascade reveals structural determinants within LBP, CD14, and TLR4-MD2 for efficient LPS recognition and transfer. Immunity 46:38–50. doi:10.1016/j.immuni.2016.11.007. 27986454

[40] Man SM, Karki R, Briard B, Burton A, Gingras S, Pelletier S, Kanneganti TD 2017 Differential roles of caspase-1 and caspase-11 in infection and inflammation. Sci Rep 7:45126. doi:10.1038/srep45126. 28345580 PMC5366862

[41] Kagan JC, Su T, Horng T, Chow A, Akira S, Medzhitov R 2008 TRAM couples endocytosis of Toll-like receptor 4 to the induction of interferon-beta. Nat Immunol 9:361–368. doi:10.1038/ni1569. 18297073 PMC4112825

